# Supplementary material for: Norepinephrine Signals Through Astrocytes To Modulate Synapses
Source: bioRxiv. 2024 May 22:2024.05.21.595135. Preprint. [Version 2] doi: 10.1101/2024.05.21.595135 (PMC11142048; doi:10.1101/2024.05.21.595135)
Supplement: Supplement 1 [file NIHPP2024.05.21.595135v2-supplement-1.pdf]

bioRxiv preprint doi: https://doi.org/10.1101/2024.05.21.595135; this version posted May 21, 2024. The copyright holder for this preprint (which was not certified by peer review) is the author/funder, who has granted bioRxiv a license to display the preprint in perpetuity. It is made available under aCC-BY 4.0 International license.

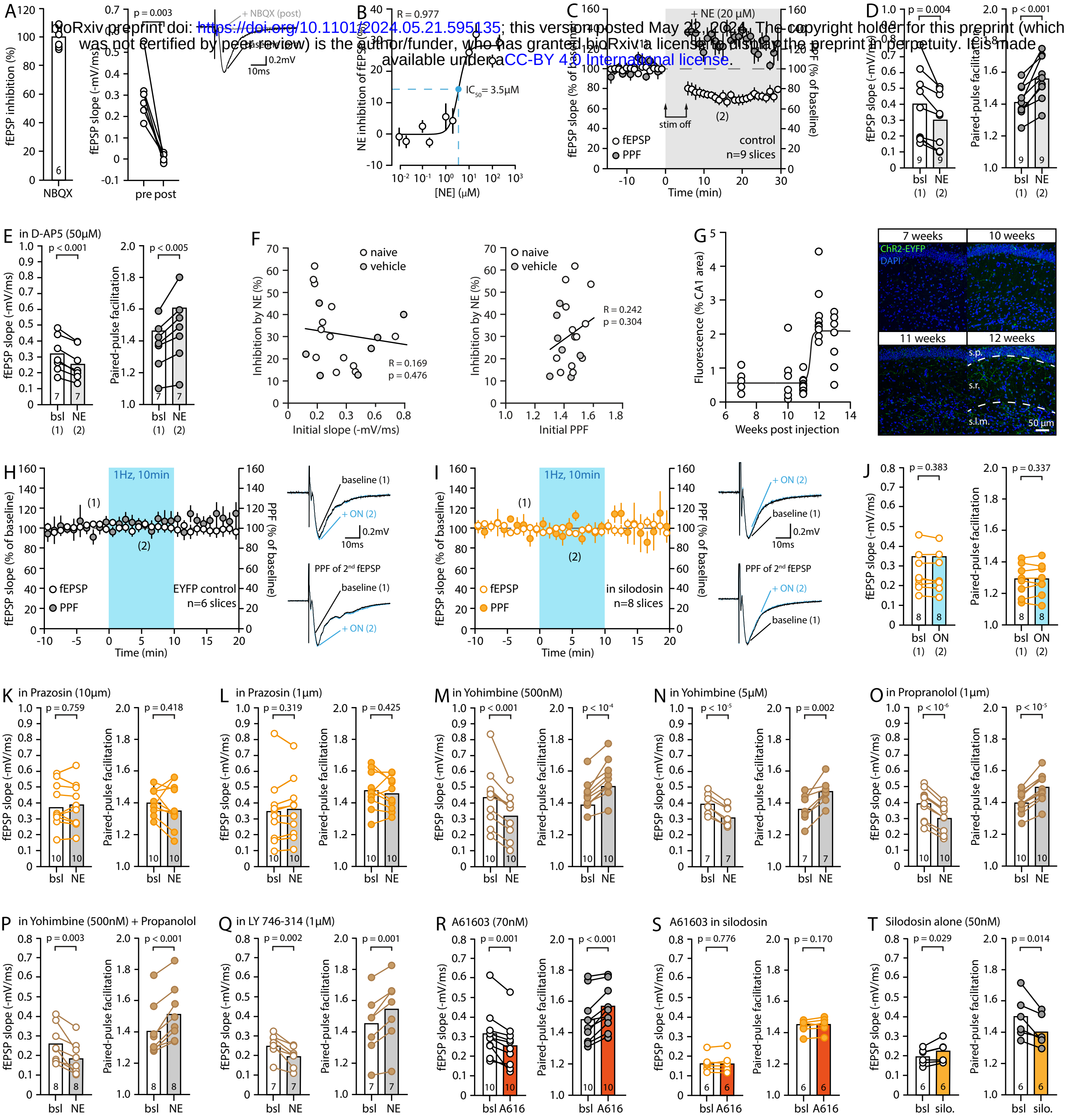

SupFigure 1

bioRxiv preprint doi: https://doi.org/10.1101/2024.05.21.595135; this version posted May 22, 2024. The copyright holder for this preprint (which was not certified by peer review) is the author/funder, who has granted bioRxiv a license to display the preprint in perpetuity. It is made available under aCC-BY 4.0 International license.

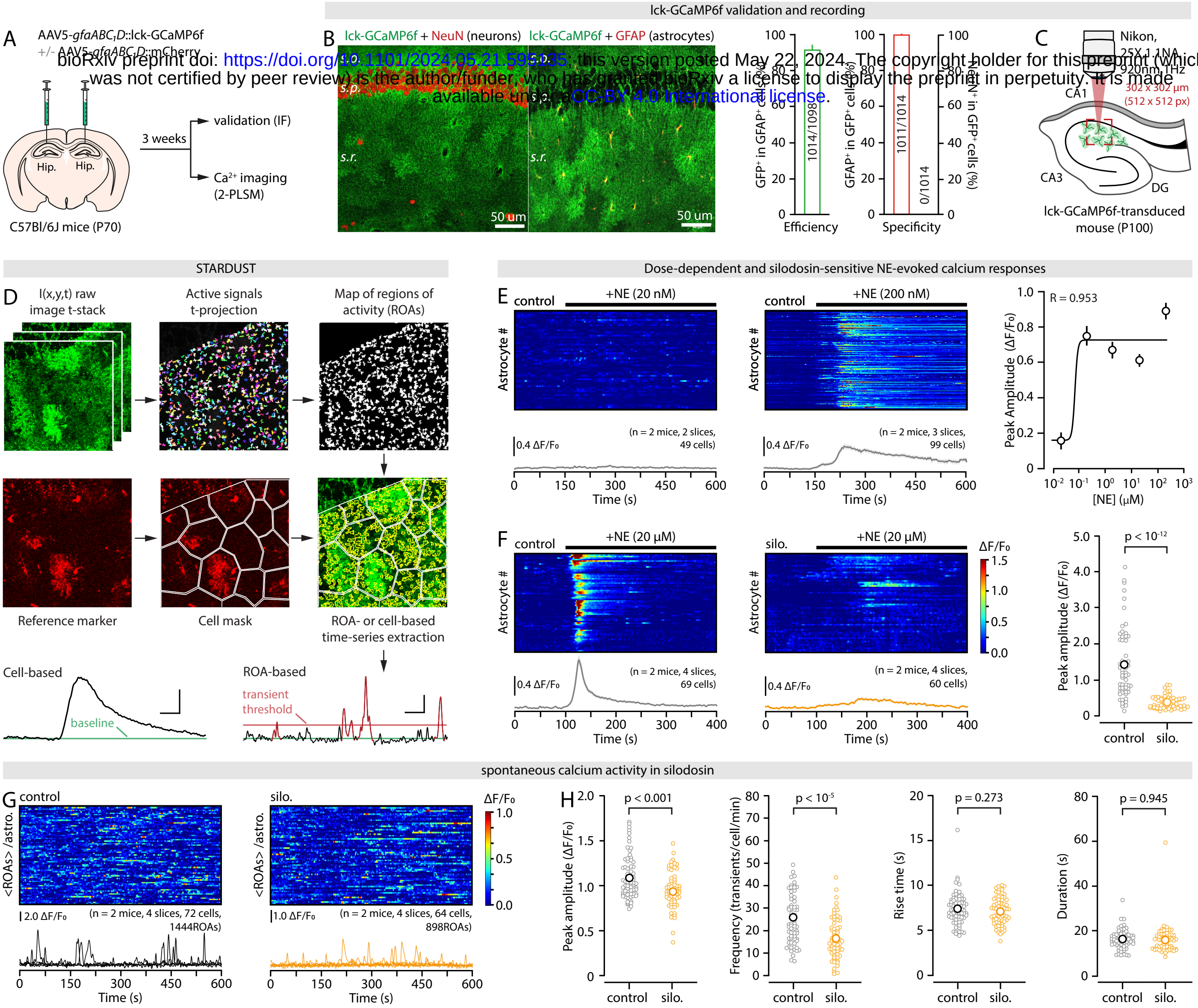

SupFigure 2

bioRxiv preprint doi: https://doi.org/10.1101/2024.05.21.595135; this version posted May 22, 2024. The copyright holder for this preprint (which was not certified by peer review) is the author/funder, who has granted bioRxiv a license to display the preprint in perpetuity. It is made available under aCC-BY 4.0 International license.

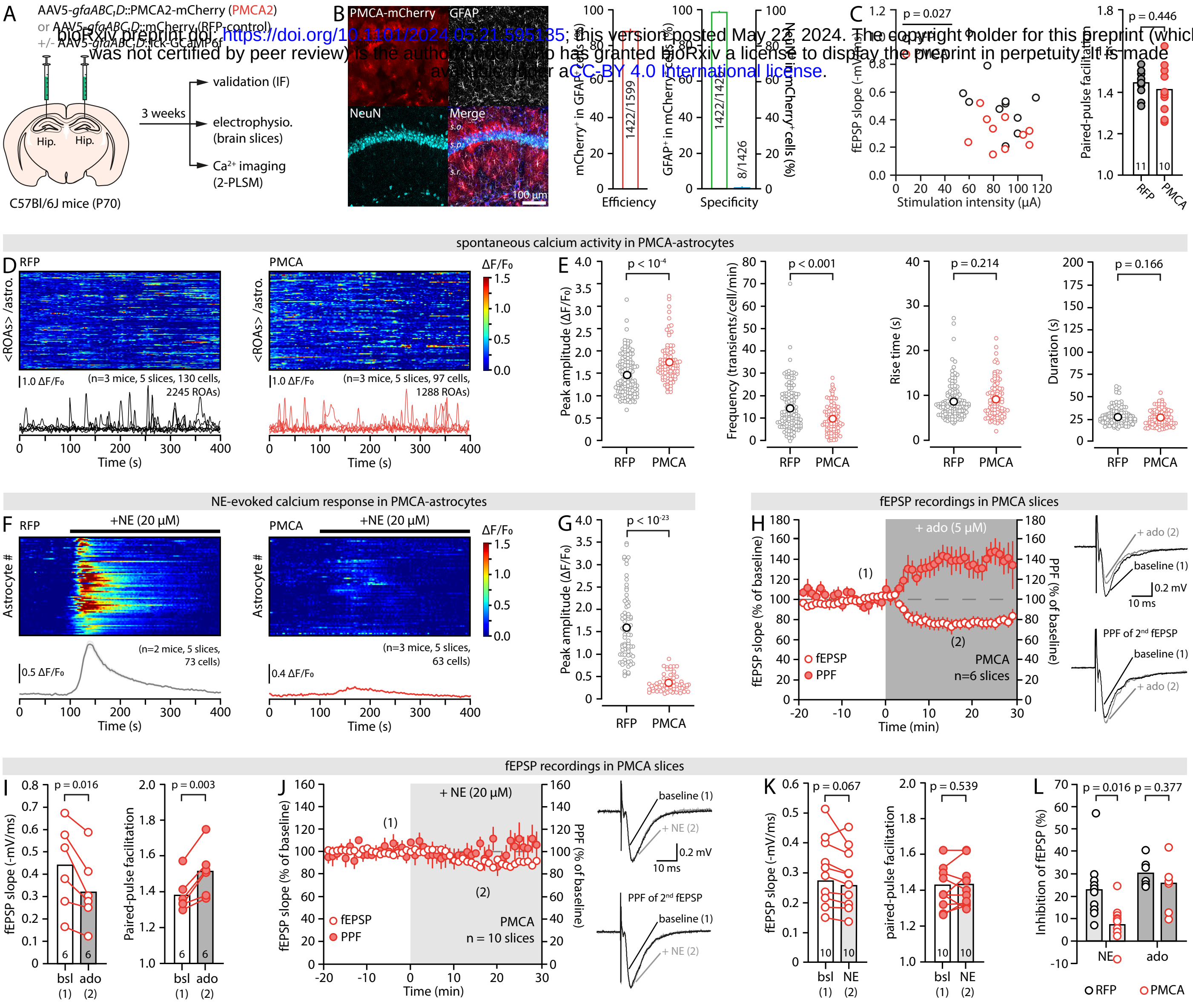

SupFigure 3

bioRxiv preprint doi: https://doi.org/10.1101/2024.05.21.595135; this version posted May 22, 2024. The copyright holder for this preprint (which was not certified by peer review) is the author/funder, who has granted bioRxiv a license to display the preprint in perpetuity. It is made available under aCC-BY 4.0 International license.

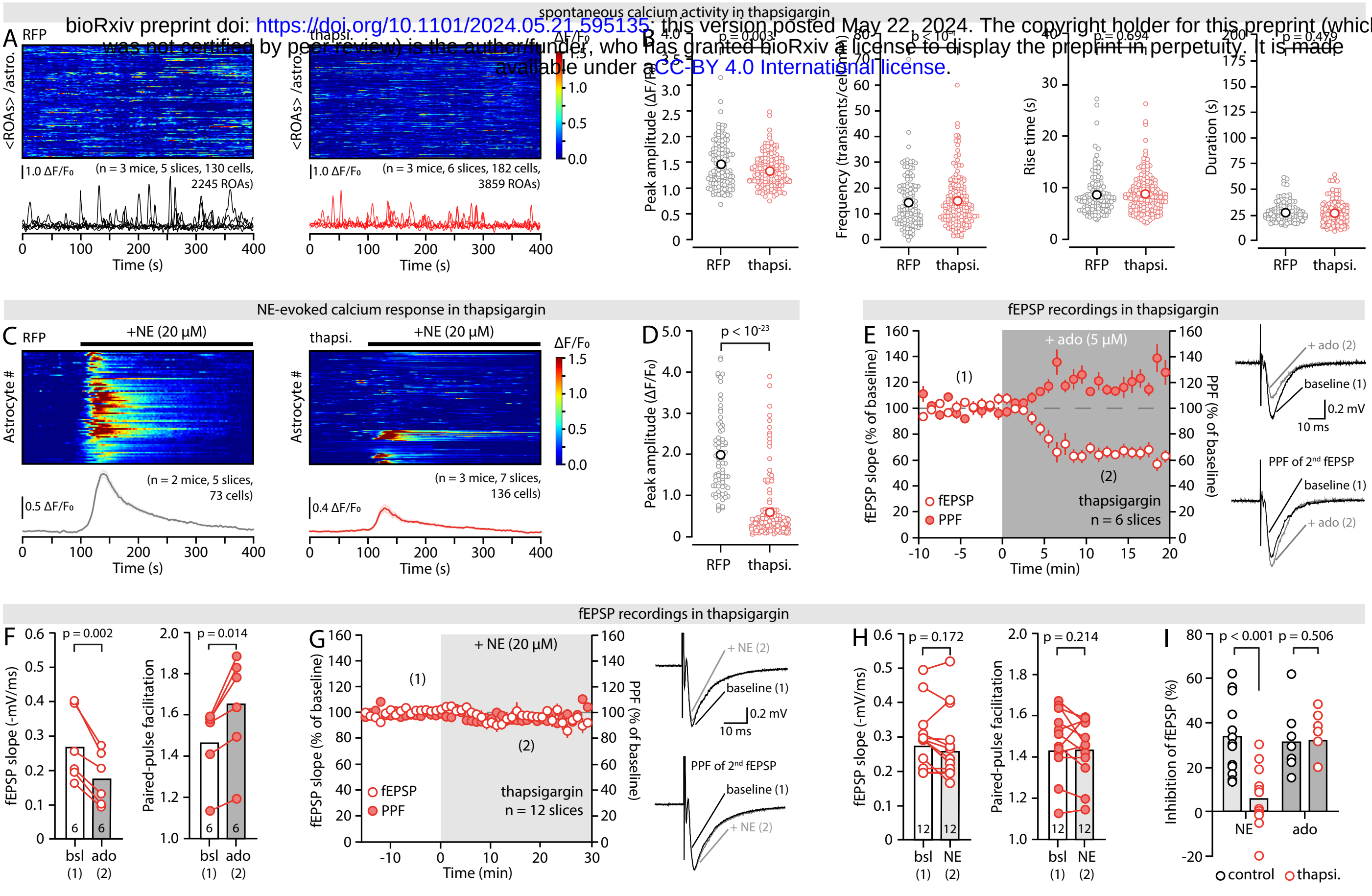

SupFigure 4

bioRxiv preprint doi: https://doi.org/10.1101/2024.05.21.595135; this version posted May 22, 2024. The copyright holder for this preprint (which was not certified by peer review) is the author/funder, who has granted bioRxiv a license to display the preprint in perpetuity. It is made available under aCC-BY 4.0 International license.

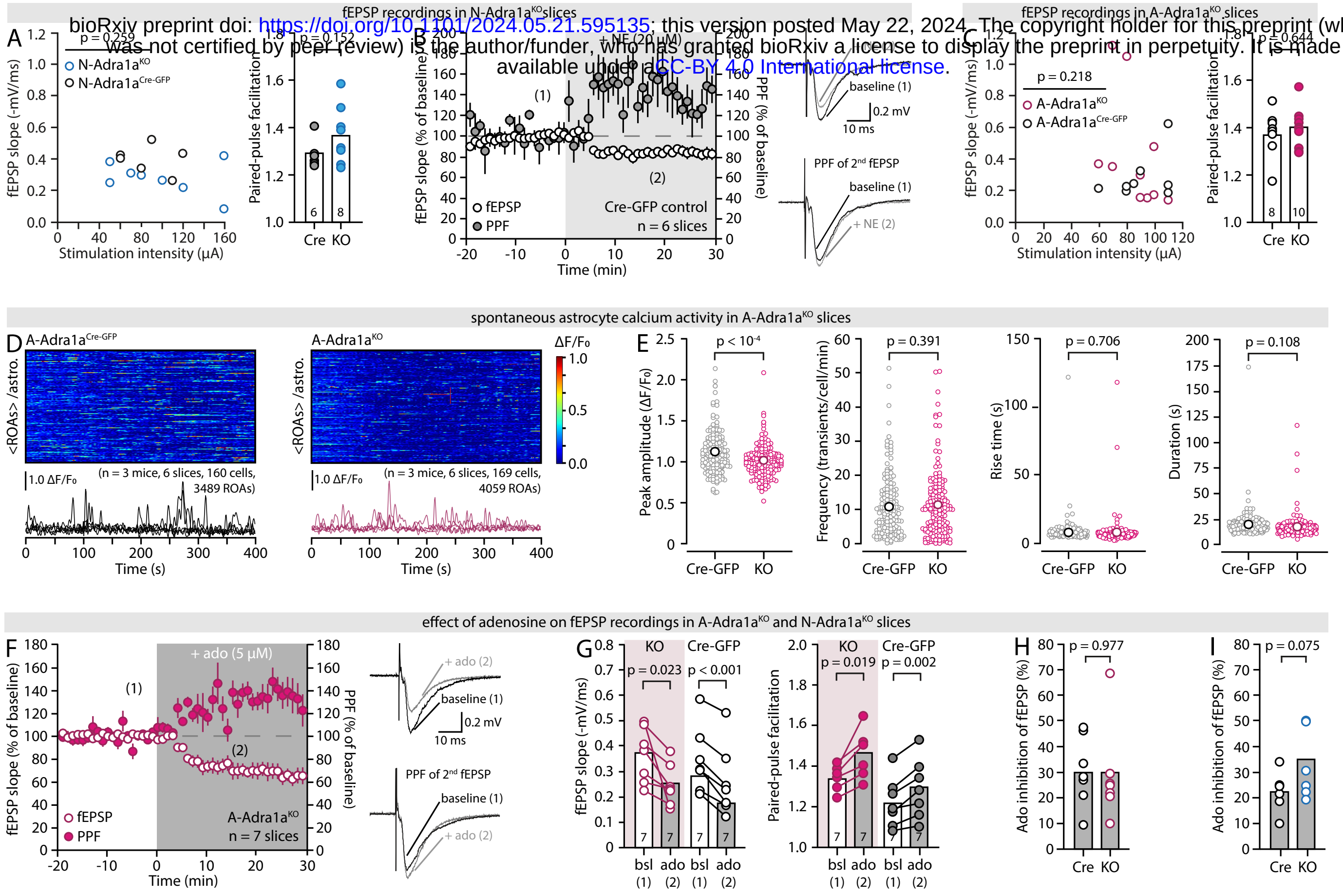

SupFigure 5

bioRxiv preprint doi: https://doi.org/10.1101/2024.05.21.595135; this version posted May 22, 2024. The copyright holder for this preprint (which was not certified by peer review) is the author/funder, who has granted bioRxiv a license to display the preprint in perpetuity. It is made available under aCC-BY 4.0 International license.

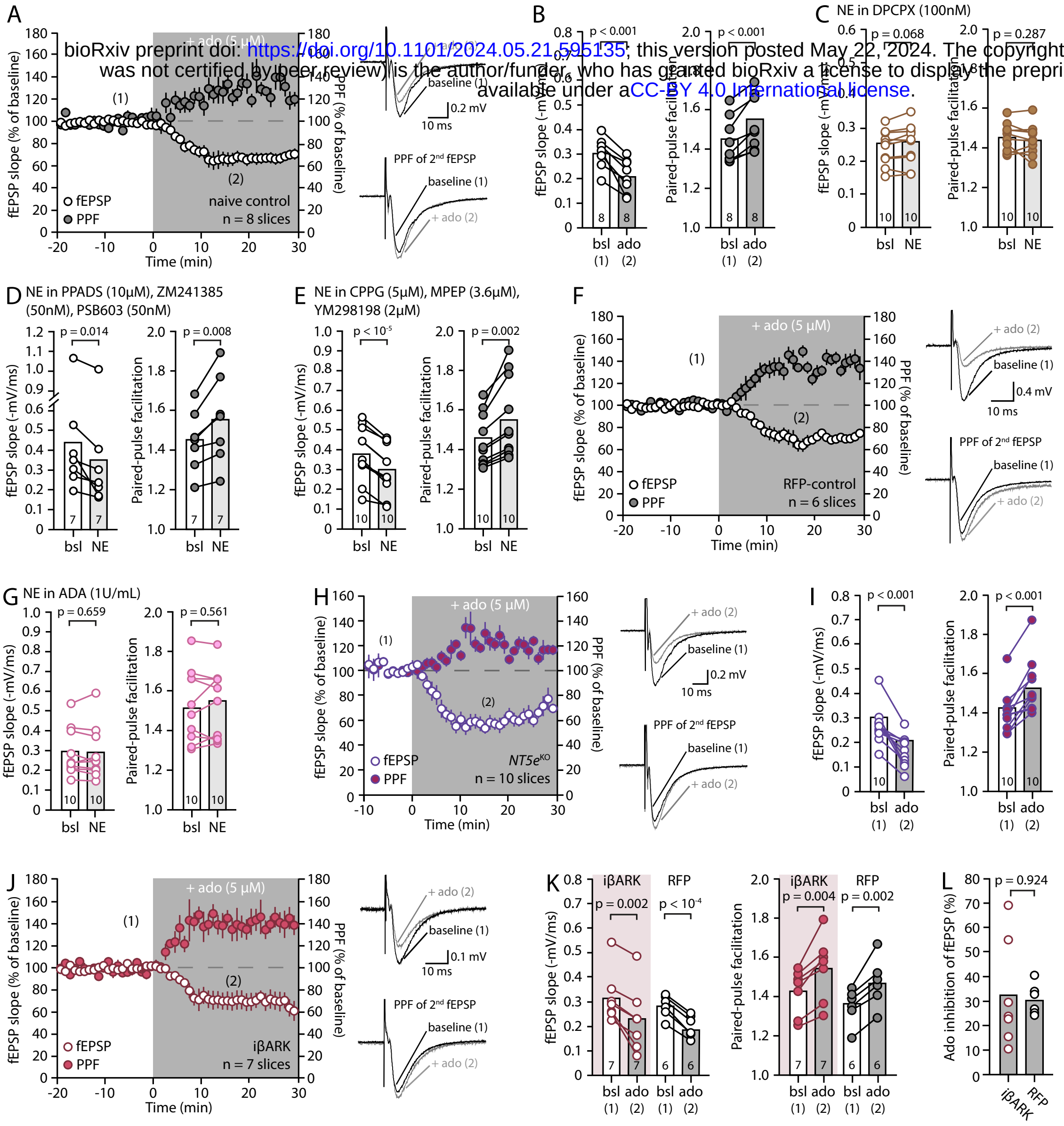

SupFigure 6

bioRxiv preprint doi: https://doi.org/10.1101/2024.05.21.595135; this version posted May 22, 2024. The copyright holder for this preprint (which was not certified by peer review) is the author/funder, who has granted bioRxiv a license to display the preprint in perpetuity. It is made available under aCC-BY 4.0 International license.

Supplementary Figure Legends

SupFig. 1: Effect of NE on the AMPAR-mediated fEPSPs

(A) Pairwise and summary quantification of the effect of the AMPAR antagonist NBQX (10µM) on fEPSPs and representative traces. **(B)** Dose-response curve of the inhibitory effect of 20µM NE on fEPSP slope (n = 5 to 7 slices per concentration). **(C,D)** Time course and pairwise quantification of the effect of NE on fEPSP and PPF with stimulations of Schaffer collaterals paused for five minutes at the onset of NE application. **(E)** Pairwise quantification of the effect of 20µM NE on fEPSP and PPF in the presence of the NMDAR antagonist D-AP5 (50µM). **(F)** Plots of the inhibitory effect of NE as a function and the initial fEPSPs slope (left) or initial PPF (right) across control conditions. The naïve and vehicle condition (DMSO, 0.01%) are denoted separately, but the linear regression, correlation coefficient and p-values are shown for the control condition as a whole. **(G)** Quantification and representative images of ChR2-eYFP expression of LC-NE projections in the hippocampal CA1 at 7, 10, 11, 12 and 13 weeks (n = 6-12 slices, from 1-2 animals). **(H)** Time course and representative traces showing the effect of the optical stimulation of LC-NE fibers on fEPSPs and PPF in EYFP-control slices. **(I,J)** Time course, representative traces and pairwise quantification showing the effect of the optical stimulation of LC-NE fibers on fEPSPs and PPF in the presence of silodosin (50nM). **(K-Q)** Pairwise quantification of the effect of 20 µM NE on fEPSP slope and PPF in prazosin (α1-AR antagonist, (K,L)), yohimbine (α2-AR antagonist, (M,N)), propranolol (β antagonist, (O)), yohimbine and propranolol (P), and LY746-314 (α1B-AR antagonist, (Q)) at indicated concentrations. **(R,S)** Pairwise quantification showing the inhibitory effect of the α1A-AR agonist A61603 (70nM) on fEPSP and PPF (R) and its blocked by silodosin (S). **(T)** Pairwise quantification showing the potentiating effect of silodosin perfusion (50nM) on fEPSP and inhibitory effect on PPF.

SupFig. 2: Imaging and analysis of astrocyte Ca^{2+} responses to NE and α1A-AR pharmacology

(A) Schematic of micro-injections for lck-GCaMP6f expression in astrocytes in the *s. radiatum* of the CA1. **(B)** IHC of lck-GCaMP6f expression in CA1 astrocytes and quantification of efficiency and specificity. **(C)** Schematic of the 2-PSLM conditions for astrocyte lck-GCaMP6f imaging in hippocampal slices. **(D)** Overview of the STARDUST analysis workflow for ROA and cell-based timeseries analysis. **(E)** *Left,* Kymographs of whole astrocyte Ca^{2+} signals (each row represents a single cell) and average $\Delta F/F_0$ traces (± s.e.m.) across all astrocytes, in responses to the application of NE at indicated concentrations. *Right*, Dose response curve showing the peak amplitude of the astrocyte Ca^{2+} response as a function of NE concentration. **(F)** *Left,* Kymographs of whole astrocyte Ca^{2+} signals (each row represents a single cell) and average $\Delta F/F_0$ traces (± s.e.m.) across all astrocytes, in response to the application of 20µM NE in control conditions and in the presence of silodosin (50nM, α1A-AR antagonist). *Right*, Quantification of the peak astrocyte Ca^{2+} response to 20µM NE in control and silodosin. **(G)** Kymographs (each row shows the average fluorescence across ROAs of a single astrocyte) and 5 representative $\Delta F/F_0$ traces (from individual ROAs) showing spontaneous astrocyte Ca^{2+} transients in control and silodosin conditions. **(H)** Quantification of the peak amplitude, frequency, and kinetics of spontaneous astrocyte Ca^{2+} transients in control conditions and in the presence of silodosin.

bioRxiv preprint doi: https://doi.org/10.1101/2024.05.21.595135; this version posted May 22, 2024. The copyright holder for this preprint (which was not certified by peer review) is the author/funder, who has granted bioRxiv a license to display the preprint in perpetuity. It is made available under aCC-BY 4.0 International license.

SupFig. 3: CalEx blocks the effect of NE on astrocyte Ca^{2+} and synapses

(A) Schematic micro-injections to express the CalEx actuator PMCA2 (plasma membrane Ca^{2+} pump) in CA1 astrocytes. **(B)** IHC of PMCA2 expression in CA1 *s. radiatum* astrocytes and quantification of specificity and efficiency. **(C)** Plot of the stimulation intensity/fEPSP slope relationship (left, unpaired Student's *t*-test on slope/stim ratio) and summary bar graphs of PPF values (right) in RFP-control and CalEx slices at baseline. **(D,E)** Kymograph (each row shows the average fluorescence across ROAs of a single astrocyte), 5 representative $\Delta F/F_0$ traces (from individual ROAs), and quantification of the peak amplitude, frequency, and kinetics of spontaneous Ca^{2+} transients in RFP-control and CalEx slices. **(F,G)** Kymographs (each row represents a single cell), average $\Delta F/F_0$ traces (± s.e.m.) across all astrocytes, and quantification of the peak amplitude, in response to 20μM NE application in RFP-control and CalEx slices. **(H)** Time course and representative traces of the effect of adenosine on fEPSP and PPF in CalEx slices. **(I)** Pairwise quantification of the effect of adenosine on fEPSP and PPF in CalEx slices. **(J,K)** Time course, representative traces, and pairwise quantifications of the effect of NE on fEPSP and PPF in CalEx slices. **(L)** Summary bar graphs showing the inhibitory effect of NE and adenosine on fEPSP in RFP-control and CalEx slices. The p-values in (L) are from ANOVAs across iβark, CalEx, and RFP-control conditions followed by Tukey's *post-hoc* test, reflecting the fact that the RFP-control condition for the experiments shown in (L) is the same across iβark (Fig.2) and CalEx (this figure) experiments.

SupFig. 4: Thapsigargin blocks the effect of NE on astrocyte Ca^{2+} and synapses

(A,B) Kymograph (each row shows the average fluorescence across ROAs of a single astrocyte), 5 representative $\Delta F/F_0$ traces (from individual ROAs), and quantification of the peak amplitude, frequency, and kinetics of spontaneous Ca^{2+} transients in TTX alone (RFP-control) and TTX + thapsigargin conditions. All slices were obtained from animals with RFP-transduced astrocytes for cell-segmentation purposes. Thapsigargin was bath applied 20min prior to the start of recording. **(C,D)** Kymographs (each row represents a single cell), average $\Delta F/F_0$ traces (± s.e.m.) across all astrocytes, and quantification of the peak amplitude, in response to 20μM NE application in RFP-control and thapsigargin conditions. **(E,F)** Time course, representative traces, and pairwise quantification of the effect of 5μM adenosine on fEPSP and PPF in thapsigargin-treated slices. **(G,H)** Time course, representative traces, and pairwise quantifications of the effect of 20μM NE on fEPSP and PPF in thapsigargin-treated slices. **(L)** Summary of the inhibitory effect of NE and adenosine on fEPSP in control and thapsigargin-treated slices.

SupFig. 5: Ca^{2+} and synaptic recordings in A- and N-Adra^{KO} slices

(A) Plot of the stimulation intensity/fEPSP slope relationship (left, unpaired Student's *t*-test on slope/stim ratio) and summary bar graphs of PPF values (right) in N-Adra^{KO} and N-Adra$^{Cre\text{-}GFP}$ slices at baseline. **(B)** Time course and representative traces of the effect of 20μM NE on fEPSP and PPF in N-Adra$^{Cre\text{-}GFP}$ slices. **(C)** Plot of the stimulation intensity/fEPSP slope relationship (left, unpaired Student's *t*-test on slope/stim ratio) and summary bar graphs of PPF values (right) in A-Adra^{KO} and A-Adra$^{Cre\text{-}GFP}$ slices at baseline. **(D,E)** Kymograph (each row shows the average fluorescence across ROAs of a single astrocyte), 5 representative $\Delta F/F_0$ traces (from individual ROAs), and quantification of the peak amplitude, frequency, and kinetics of spontaneous Ca^{2+} transients in A-Adra^{KO} and A-Adra$^{Cre\text{-}GFP}$ slices. **(F)** Time course and representative traces of the effect of 5μM adenosine on fEPSP and PPF in A-Adra^{KO} slices. **(G)** Pairwise quantification of the effect of 5μM adenosine on fEPSP and PPF in A-Adra^{KO} and A-

bioRxiv preprint doi: https://doi.org/10.1101/2024.05.21.595135; this version posted May 22, 2024. The copyright holder for this preprint (which was not certified by peer review) is the author/funder, who has granted bioRxiv a license to display the preprint in perpetuity. It is made available under aCC-BY 4.0 International license.

Adra$^{Cre\text{-}GFP}$ slices. **(H)** Summary plot of the inhibitory effect of adenosine on fEPSP in A-Adra^{KO} and A-Adra$^{Cre\text{-}GFP}$ slices. **(I)** Summary plot of the inhibitory effect of adenosine on fEPSP in N-Adra^{KO} and N-Adra$^{Cre\text{-}GFP}$ slices.

SupFig. 6: Adenosine signaling is central to the effect of NE and preserved in NT5e^{KO} animals and across astrocyte Ca^{2+} interventions

(A,B) Time course, representative traces, and pairwise quantifications of the effect of adenosine on fEPSP and PPF in slices from naïve animals. **(C-E)** Pairwise quantification of the effect of 20μM NE on fEPSP and PPF in DPCPX (100nM, C), a cocktail of P2X and P2Y inhibitors (D), and a cocktail of mGluR inhibitors (E). **(F)** Time courses and representative traces of the effect of 5μM adenosine on fEPSP and PPF in RFP-control slices. **(G)** Pairwise quantification of the effect of 20μM NE on fEPSP and PPF in ADA (1U/mL). **(H,I)** Time course, representative traces, and pairwise quantification of the effect of 5μM adenosine on fEPSP and PPF in NT5e^{KO} slices (paired Student's *t*-tests). **(J)** Time courses and representative traces of the effect of 5μM adenosine on fEPSP and PPF in iβARK slices. (**K**) Pairwise quantification of the effect of adenosine on fEPSP and PPF in iβARK and RFP-control slices **(L)** Summary plot of the inhibitory effect of adenosine on fEPSP in iβARK and RFP-control slices. The p-value in (L) is from an ANOVA across iβark, CalEx and RFP-control conditions followed by Tukey's *post-hoc* test, reflecting the fact that the RFP-control condition for fEPSP recordings experiments is common to CalEx (SupFig.3) and iβark (Fig.2).

bioRxiv preprint doi: https://doi.org/10.1101/2024.05.21.595135; this version posted May 22, 2024. The copyright holder for this preprint (which was not certified by peer review) is the author/funder, who has granted bioRxiv a license to display the preprint in perpetuity. It is made available under aCC-BY 4.0 International license.

Supplementary Tables

Figure	Panel	Measure	Groups	Statistical test	Test value	p value
1	C	fEPSP slope	Baseline vs +NE	Paired Student's *t*-test	t=7.346	8.9E-06
		PPF	Baseline vs +NE	Paired Student's *t*-test	t=5.355	0.00017
	D	Correlation of ΔfEPSP, ΔPPF	PPF Increase, fEPSP Decrease	Pearson's Correlation	R=0.9035	$<10^{-4}$
	G	Synaptic efficacy	Baseline vs +NE	Paired Student's *t*-test	t=3.421	0.008
		Synaptic potency	Baseline vs +NE	Paired Student's *t*-test	t=0.6007	0.564
		Synaptic strength	Baseline vs +NE	Paired Student's *t*-test	t=2.313	0.049
	L	ChR2+, fEPSP slope	Baseline vs +Stim	Paired Student's *t*-test	t=3.234	0.01783
		EYFP, fEPSP slope	Baseline vs +Stim	Paired Student's *t*-test	t=1.162	0.29781
		ChR2+, PPF	Baseline vs +Stim	Paired Student's *t*-test	t=2.902	0.02726
		EYFP, PPF	Baseline vs +Stim	Paired Student's *t*-test	t=1.397	0.22125
	M	Inhibition of fEPSP	ChR2+ vs ChR2+Silodosin	ANOVA, multiple comparisons, Tukey's post-hoc test	F=11.19	0.035
			ChR2+ vs EYFP			0.0031
	N	Inhibition of fEPSP slope	Control vs Propranolol	ANOVA, multiple comparisons, Tukey's post-hoc test	F=16.34	0.1772
			Control vs Yohimbine			0.6458
			Control vs Yohimbine+Propranolol			0.9004
			Control vs LY746-314			0.4971
			Control vs Prazosin			<0.0001
			Control vs Silodosin			<0.0001
			Control vs A61603			0.027
	P	silodosin, fEPSP slope	Baseline vs +NE	Paired Student's *t*-test	t=0.6328	0.54267
		silodosin, PPF	Baseline vs +NE	Paired Student's *t*-test	t=1.010	0.33892
2	C	Initial fEPSP slope vs stimulation intensity	RFP vs IBARK	Unpaired Student's *t*-test	t=1.8454	0.081
		Initial PPF	RFP vs IBARK	Unpaired Student's *t*-test	t=0.6627	0.5159
	E	Peak amplitude	RFP vs IBARK	Permutation test	$T_{obs=}$ 0.390678721	$<10^{-4}$
		Frequency	RFP vs IBARK	Permutation test	$T_{obs=}$ 0.167221002	$<10^{-4}$
		Rise time	RFP vs IBARK	Permutation test	$T_{obs=}$	0.008

bioRxiv preprint doi: https://doi.org/10.1101/2024.05.21.595135; this version posted May 22, 2024. The copyright holder for this preprint (which was not certified by peer review) is the author/funder, who has granted bioRxiv a license to display the preprint in perpetuity. It is made available under aCC-BY 4.0 International license.

					1.012163116	
		Duration	RFP vs IBARK	Permutation test	T_{obs}= 2.257451108	0.102
	G	Amplitude	RFP vs IBARK	Permutation test	T_{obs}= 1.195065825	$<10^{-4}$
	J	iβARK, fEPSP slope	Baseline vs +NE	Paired Student's *t*-test	t=1.995	0.08113
		RFP-control, fEPSP slope	Baseline vs +NE	Paired Student's *t*-test	t=5.249	0.00037
		iβARK, PPF	Baseline vs +NE	Paired Student's *t*-test	t=1.826	0.10533
		RFP-control, PPF	Baseline vs +NE	Paired Student's *t*-test	t=12.10	2.7E-07
	K	Inhibition of fEPSP slope by NE	RFP vs IBARK*	ANOVA, multiple comparisons, Tukey's post-hoc test	F=5.449	0.0038
	L	Correlation of fEPSP and Ca^{2+} inhibition by NE	RFP, IBARK, CalEx, Thapsigargin	Pearson's Correlation	R=0.9727	0.027
3	C	N-Adra^{KO}, sorted cell band intensity	N-Adra^{KO} GFP+ vs GFP-	Unpaired Student's *t*-test	t=3.318	0.03
	E	N-Adra^{KO}, fEPSP slope	Baseline vs +NE (N-Adra^{KO})	Paired Student's *t*-test	t=5.753	0.0007
		N-Adra^{Control}, fEPSP slope	Baseline vs +NE (N-Adra^{Control})	Paired Student's *t*-test	t=5.394	0.00202
		N-Adra^{KO}, PPF	Baseline vs +NE (N-Adra^{KO})	Paired Student's *t*-test	t=10.79	1.3E-05
		N-Adra^{Control}, PPF	Baseline vs +NE (N-Adra^{Control})	Paired Student's *t*-test	t=5.883	0.00296
	F	Inhibition of fEPSP slope by NE	N-Adra^{KO} vs N-Adra^{Control}	Unpaired Student's *t*-test	t=1.297	0.219
	I	Adra1a band intensity, bulk samples	A-Adra^{KO} vs A-Adra^{Control}	Unpaired Student's *t*-test	t=2.689	0.04333
		Adra1a band intensity, sorted cells	A-Adra^{KO} GFP+ vs GFP-	Unpaired Student's *t*-test	t=3.619	0.02237
	K	Peak Ca^{2+} amplitude	A-Adra^{KO} vs A-Adra^{Control}	Permutation test	T_{obs}= 1.161589806	$<1x10^{-19}$
	N	A-Adra^{KO}, fEPSP slope	Baseline vs +NE (A-Adra^{KO})	Paired Student's *t*-test	t=0.9469	0.94687
		A-Adra^{Control}, fEPSP slope	Baseline vs +NE (A-Adra^{Control})	Paired Student's *t*-test	t=5.074	0.00144

bioRxiv preprint doi: https://doi.org/10.1101/2024.05.21.595135; this version posted May 22, 2024. The copyright holder for this preprint (which was not certified by peer review) is the author/funder, who has granted bioRxiv a license to display the preprint in perpetuity. It is made available under aCC-BY 4.0 International license.

		A-Adra^{KO}, PPF	Baseline vs +NE (A-Adra^{KO})	Paired Student's *t*-test	t=0.2201	0.22007
		A-Adra^{Control}, PPF	Baseline vs +NE (A-Adra^{Control})	Paired Student's *t*-test	t=9.215	3.7E-05
	O	Inhibition of fEPSP slope by NE	A-Adra^{KO} vs A-Adra^{Control}	Unpaired Student's *t*-test	t=4.453	0.21914
	P	Remaining change in fEPSP slope	A-Adra^{KO} vs A-Adra^{Control}	Unpaired Student's *t*-test	t=4.453	5.7E-06
4	A	Inhibition of fEPSP slope by NE	Control vs P2R/A2R	ANOVA, multiple comparisons, Tukey's post-hoc test	F=20.12	0.1942
			Control vs mGluR			0.1419
			Control vs NT5e^{KO}			<0.0001
			Control vs ADA			<0.0001
			Control vs CPT			<0.0001
			Control vs DPCPX			<0.0001
	C	fEPSP slope	Baseline vs +NE	Paired Student's *t*-test	t=1.369	0.20426
		PPF	Baseline vs +NE	Paired Student's *t*-test	t=0.1102	0.91468
	G	Inhibition of fEPSP slope by ado	CA1-Ado1^{KD} vs CA3-Ado1^{KD}	Unpaired Student's *t*-test	t=2.449	0.00914
	I	CA1-Ado^{KD}, fEPSP slope	Baseline vs +NE	Paired Student's *t*-test	t=5.361	0.00173
		CA3-Ado^{KD}, fEPSP slope	Baseline vs +NE	Paired Student's *t*-test	t=5.233	0.00028
	J	Inhibition of fEPSP slope by NE	CA1-Ado1^{KD} vs CA3-Ado1^{KD}	Unpaired Student's *t*-test	t=4.484	0.00033
	L	fEPSP slope	Baseline vs +NE	Paired Student's *t*-test	t=0.5245	0.61262
		PPF	Baseline vs +NE	Paired Student's *t*-test	t=0.3189	0.75708
	N	Inhibition of fEPSP slope by Ado	Naïve vs NT5e^{KO}	Unpaired Student's *t*-test	t=1.118	0.28004
S1	A	fEPSP slope	Baseline vs +NBQX	Paired Student's *t*-test	t=5.278	0.00325
	D	fEPSP slope	Baseline vs +NE	Paired Student's *t*-test	t=3.984	0.00404
		PPF	Baseline vs +NE	Paired Student's *t*-test	t=5.679	0.00075
	E	fEPSP slope	Baseline vs +NE	Paired Student's *t*-test	t=8.309	0.00016
		PPF	Baseline vs +NE	Paired Student's *t*-test	t=4.371	0.00471
	F	Correlation of initial slope	Naïve vs vehicle	Pearson's Correlation	R=0.169	0.476

bioRxiv preprint doi: https://doi.org/10.1101/2024.05.21.595135; this version posted May 22, 2024. The copyright holder for this preprint (which was not certified by peer review) is the author/funder, who has granted bioRxiv a license to display the preprint in perpetuity. It is made available under aCC-BY 4.0 International license.

		and inhibition of fEPSP by NE				
		Correlation of initial PPF and inhibition of fEPSP by NE	Naïve vs vehicle	Pearson's Correlation	R=0.242	0.304
	J	fEPSP slope	Baseline vs +Stim	Paired Student's *t*-test	t=0.9303	0.38336
		PPF	Baseline vs +Stim	Paired Student's *t*-test	t=1.030	0.3374
	K	fEPSP slope	Baseline vs +NE	Paired Student's *t*-test	t=0.3156	0.75949
		PPF	Baseline vs +NE	Paired Student's *t*-test	t=0.4184	0.41836
	L	fEPSP slope	Baseline vs +NE	Paired Student's *t*-test	t=1.053	0.31991
		PPF	Baseline vs +NE	Paired Student's *t*-test	t=0.8345	0.42557
	M	fEPSP slope	Baseline vs +NE	Paired Student's *t*-test	t=5.085	0.00066
		PPF	Baseline vs +NE	Paired Student's *t*-test	t=7.591	3.4E-05
	N	fEPSP slope	Baseline vs +NE	Paired Student's *t*-test	t=14.29	7.4E-06
		PPF	Baseline vs +NE	Paired Student's *t*-test	t=5.179	0.00206
	O	fEPSP slope	Baseline vs +NE	Paired Student's *t*-test	t=14.73	1.3E-07
		PPF	Baseline vs +NE	Paired Student's *t*-test	t=10.56	2.3E-06
	P	fEPSP slope	Baseline vs +NE	Paired Student's *t*-test	t=4.477	0.00288
		PPF	Baseline vs +NE	Paired Student's *t*-test	t=6.187	0.00045
	Q	fEPSP slope	Baseline vs +NE	Paired Student's *t*-test	t=5.326	0.00178
		PPF	Baseline vs +NE	Paired Student's *t*-test	t=5.722	0.00123
	R	fEPSP slope	Baseline vs +NE	Paired Student's *t*-test	t=4.684	0.00115
		PPF	Baseline vs +NE	Paired Student's *t*-test	t=5.186	0.00057
	S	fEPSP slope	Baseline vs +NE	Paired Student's *t*-test	t=0.3004	0.77643
		PPF	Baseline vs +NE	Paired Student's *t*-test	t=1.600	0.17043
	T	fEPSP slope	Baseline vs +NE	Paired Student's *t*-test	t=3.024	0.02926
		PPF	Baseline vs +NE	Paired Student's *t*-test	t=3.665	0.01452
S2	F	Peak amplitude	Control vs Silodosin	Unpaired Student's *t*-test	T_{obs}= 1.035840725	$<10^{-12}$

bioRxiv preprint doi: https://doi.org/10.1101/2024.05.21.595135; this version posted May 22, 2024. The copyright holder for this preprint (which was not certified by peer review) is the author/funder, who has granted bioRxiv a license to display the preprint in perpetuity. It is made available under aCC-BY 4.0 International license.

	H	Peak amplitude	Control vs Silodosin	Permutation test	T_{obs}= 0.138244803	<0.001
		Frequency	Control vs Silodosin	Permutation test	T_{obs}= 5.599700638	$<10^{-5}$
		Rise time	Control vs Silodosin	Permutation test	T_{obs}= 0.314160344	0.273
		Duration	Control vs Silodosin	Permutation test	T_{obs}= 0.254129255	0.945
S3	C	Initial Stimulation vs Intensity	RFP-Control vs PMCA	Unpaired Student's *t*-test	t=2.393	0.027
		Initial PPF	RFP-Control vs PMCA	Unpaired Student's *t*-test	t=0.7774	0.446
	E	Peak amplitude	RFP-Control vs PMCA	Permutation test	T_{obs}= -0.277103985	$<10^{-4}$
		Frequency	RFP-Control vs PMCA	Permutation test	T_{obs}= 0.088466898	<0.001
		Rise time	RFP-Control vs PMCA	Permutation test	T_{obs}= -0.530999365	0.214
		Duration	RFP-Control vs PMCA	Permutation test	T_{obs}= 1.360463118	0.166
	G	Peak amplitude	RFP-Control vs PMCA	Permutation test	T_{obs}= 1.240199315	$<10^{-23}$
	I	fEPSP slope	Baseline vs +Ado	Paired Student's *t*-test	t=3.588	0.01574
		PPF	Baseline vs +Ado	Paired Student's *t*-test	t=5.333	0.00311
	K	fEPSP slope	Baseline vs +NE	Paired Student's *t*-test	t=2.085	0.06677
		PPF	Baseline vs +NE	Paired Student's *t*-test	t=0.6386	0.53898
	L	Inhibition of fEPSP slope by NE	RFP-Control vs PMCA*	ANOVA, multiple comparisons, Tukey's post-hoc test	F=5.449	0.0164
		Inhibition of fEPSP slope by Ado	RFP-Control vs PMCA*	ANOVA, multiple comparisons, Tukey's post-hoc test	F=0.5146	0.377
S4	B	Peak amplitude	RFP-Control vs Thapsigargin	Permutation test	T_{obs}= 0.13724117	0.003
		Frequency	RFP-Control vs Thapsigargin	Permutation test	T_{obs}= 0.093735278	$<10^{-4}$
		Rise time	RFP-Control vs Thapsigargin	Permutation test	T_{obs}= -0.176077519	0.694
		Duration	RFP-Control vs Thapsigargin	Permutation test	T_{obs}= -0.843263277	0.479
	D	Peak amplitude	RFP-Control vs Thapsigargin	Permutation test	T_{obs}= 1.004797792	$<10^{-23}$

bioRxiv preprint doi: https://doi.org/10.1101/2024.05.21.595135; this version posted May 22, 2024. The copyright holder for this preprint (which was not certified by peer review) is the author/funder, who has granted bioRxiv a license to display the preprint in perpetuity. It is made available under aCC-BY 4.0 International license.

	F	fEPSP	Baseline vs +Ado	Paired Student's *t*-test	t=5.928	0.00195
		PPF	Baseline vs +Ado	Paired Student's *t*-test	t=3.679	0.0143
	H	fEPSP	Baseline vs +NE	Paired Student's *t*-test	t=1.459	0.17237
		PPF	Baseline vs +NE	Paired Student's *t*-test	t=1.317	0.21449
	I	Inhibition of fEPSP by NE	Naïve Control vs Thapsigargin	Unpaired Student's *t*-test	t=4.138	0.00026
		Inhibition of PPF by Ado	Naïve Control vs Thapsigargin	Unpaired Student's *t*-test	t=0.6853	0.50616
S5	A	fEPSP slope vs stimulation intensity	N-Adra^{KO} vs N-Adra^{Control}	Unpaired Student's *t*-test	t=1.323	0.259
		Initial PPF	N-Adra^{KO} vs N-Adra^{Control}	Unpaired Student's *t*-test	t=1.529	0.15216
	C	fEPSP slope vs stimulation intensity	A-Adra^{KO} vs A-Adra^{Control}	Unpaired Student's *t*-test	t=1.260	0.218
		Initial PPF	A-Adra^{KO} vs A-Adra^{Control}	Unpaired Student's *t*-test	t=0.4697	0.64493
	E	Peak amplitude	A-Adra^{KO} vs A-Adra^{Control}	Permutation test	T_{obs}= 0.108641124	$<10^{-4}$
		Frequency	A-Adra^{KO} vs A-Adra^{Control}	Permutation test	T_{obs}= 0.01615977	0.391
		Rise time	A-Adra^{KO} vs A-Adra^{Control}	Permutation test	T_{obs}= 0.450011548	0.706
		Duration	A-Adra^{KO} vs A-Adra^{Control}	Permutation test	T_{obs}= 2.865231823	0.108
	G	fEPSP slope	Baseline vs Adenosine (A-Adra^{KO})	Paired Student's *t*-test	t=3.018	0.02345
		fEPSP slope	Baseline vs Adenosine (A-Adra^{Control})	Paired Student's *t*-test	t=8.333	0.00016
		PPF	Baseline vs Adenosine (A-Adra^{KO})	Paired Student's *t*-test	t=3.166	0.01941
		PPF	Baseline vs Adenosine (A-Adra^{Control})	Paired Student's *t*-test	t=4.966	0.00254
	H	fEPSP slope	A-Adra^{KO} vs A-Adra^{Control}	Unpaired Student's *t*-test	t=0.02940	0.97703
	I	PPF	N-Adra^{KO} vs N-Adra^{Control}	Unpaired Student's *t*-test	t=1.964	0.07531

bioRxiv preprint doi: https://doi.org/10.1101/2024.05.21.595135; this version posted May 22, 2024. The copyright holder for this preprint (which was not certified by peer review) is the author/funder, who has granted bioRxiv a license to display the preprint in perpetuity. It is made available under aCC-BY 4.0 International license.

S6	B	fEPSP slope	Baseline vs +Ado	Paired Student's *t*-test	t=5.759	0.00069
		PPF	Baseline vs +Ado	Paired Student's *t*-test	t=5.436	0.00097
	C	fEPSP slope	Baseline vs +NE	Paired Student's *t*-test	t=2.077	0.06758
		PPF	Baseline vs +NE	Paired Student's *t*-test	t=1.131	0.28724
	D	fEPSP slope	Baseline vs +NE	Paired Student's *t*-test	t=3.428	0.01401
		PPF	Baseline vs +NE	Paired Student's *t*-test	t=3.900	0.00798
	E	fEPSP slope	Baseline vs +NE	Paired Student's *t*-test	t=9.481	5.6E-06
		PPF	Baseline vs +NE	Paired Student's *t*-test	t=4.247	0.00215
	G	fEPSP slope	Baseline vs +NE	Paired Student's *t*-test	t=0.4561	0.65935
		PPF	Baseline vs +NE	Paired Student's *t*-test	t=0.6035	0.56111
	I	fEPSP slope	Baseline vs +Ado	Paired Student's *t*-test	t=5.891	0.00023
		PPF	Baseline vs +Ado	Paired Student's *t*-test	t=5.535	0.00036
	K	IBARK, fEPSP slope	Baseline vs +Ado (IBARK)	Paired Student's *t*-test	t=5.023	0.0024
		RFP-control, fEPSP slope	Baseline vs + Ado (RFP-Control)	Paired Student's *t*-test	t=13.66	3.8E-05
		IBARK, PPF	Baseline vs +Ado (IBARK)	Paired Student's *t*-test	t=4.493	0.00413
		RFP-control, PPF	Baseline vs + Ado (RFP-Control)	Paired Student's *t*-test	t=5.859	0.00205
	L	Inhibition of fEPSP by Ado	RFP-Control vs IBARK*	ANOVA, multiple comparisons, Tukey's post-hoc test	F=0.2971	0.9239

Table S1: Summary of statistical tests, p values, and statistical test values for all experiments illustrated in figures. * indicates that experiments were compared across RFP-Control, iβARK, and PMCA conditions.

bioRxiv preprint doi: https://doi.org/10.1101/2024.05.21.595135; this version posted May 22, 2024. The copyright holder for this preprint (which was not certified by peer review) is the author/funder, who has granted bioRxiv a license to display the preprint in perpetuity. It is made available under aCC-BY 4.0 International license.

**Target Gene**	**Primer Sequence**
Adora1	Forward: CCACCATTATCTGGCTCCCAT<br>Reverse: GCTGAGTCACCACTGTCTTGT
Adra1a	Forward: AGCTAACCATTTCAGCAAAGA<br>Reverse: CAAGATCACCCCAAGTAGAAT
Actb	Forward: GGCTGTATTCCCCTCCATCG<br>Reverse: CCAGTTGGTAACAATGCATGT
Cre	Forward: AGGCATAAATGGCAGAGTGG<br>Reverse (mutant): CATGTCCATCAGGTTCTTGC<br>Reverse (wildtype): TGGAGCTGGAGGTGGATGAT
Nt5e	Forward (mutant): GTTTTGATGCGTTCTGCAAG<br>Forward (wildtype): GCTACTTCCATTTGTCACGTCC<br>Reverse: TACCGTTGGCTGACCTTTGT

Table S2: Primer sequences used for genotyping and validation of recombination. All primers listed in 5’ to 3’.

**Target**	**Host**	**Vendor**	**Product Number**
GFAP	Chicken	Abcam	AB4674
NeuN	Guinea Pig	Millipore Sigma	ABN90
Tyrosine Hydroxylase	Rabbit	ThermoFisher	PA5-85167
GFP	Rabbit	Abcam	AB290
RFP	Rabbit	Abcam	AB62341

Table S3: Primary antibodies used for immunofluorescence experiments.

**Host**	**Alexa Fluorophore**	**Vendor**	**Product Number**
Goat-anti-Rabbit	488	ThermoFisher	A-11034
Goat-anti-Rabbit	568	ThermoFisher	A-11011
Goat-anti-Chicken	488	ThermoFisher	A-11039
Goat-anti-Chicken	647	ThermoFisher	A-32933
Goat-anti-Guinea Pig	647	ThermoFisher	A-21450

Table S4: Secondary antibodies used for immunofluorescence experiments.
